# In vitro and in vivo characterization of a novel West Nile virus lineage 2 strain

**DOI:** 10.1038/s44298-024-00070-0

**Published:** 2024-11-25

**Authors:** Imke Visser, Eleanor M. Marshall, Gianfilippo Agliani, Melanie Rissmann, Judith M. A. van den Brand, Marion P. G. Koopmans, Barry Rockx

**Affiliations:** 1 Department of Viroscience, Erasmus MC, Rotterdam, the Netherlands; 2 Division of Pathology, Faculty of Veterinary Medicine, Utrecht University, Utrecht, the Netherlands

**Keywords:** Virology, Viral pathogenesis, West nile virus

## Abstract

Over recent decades, West Nile virus (WNV) has continued to expand its geographical range, emerging in previously non-endemic areas, including northern Europe. In Europe, WNV lineage 2 strains are most prevalent and cause sporadic outbreaks of WNV disease in humans each transmission season. Here, we assessed the virulence of a newly emerged WNV lineage 2 strain that was isolated in the Netherlands in 2020 (WNV-NL20) and caused several cases of West Nile disease in humans and used a WNV lineage 2 strain related to major outbreaks of neuroinvasive disease in humans in central and south-eastern Europe in 2010 (WNV-578/10) as a reference. Infection of primary human cells of the blood-brain barrier in vitro did not show major differences in replication kinetics between WNV-578/10 and WNV-NL20. Experimental infection of mice showed that both WNV strains induced significant weight loss, neurological signs, and lethal disease. Neurological involvement was confirmed for both WNV strains by the presence of infectious virus and viral antigen in the brain. In conclusion, we show that the recent WNV-NL20 strain that emerged in the Netherlands is neurovirulent in mice. The use of in vitro and in vivo models to characterize the pathogenesis of emerging WNV strains may aid in predicting the neurovirulence of WNV infections in humans during potential future outbreaks.

## Introduction

Over the past two decades, there has been a large increase in epidemic arthropod-borne (arbo) viral diseases, such as Dengue virus, Chikungunya virus, Yellow fever virus, Zika virus, and West Nile virus (WNV)^1^. The global emergence of arboviruses is partly the result of multiple anthropological factors such as urbanization, increased travel, and international trade^2^. Moreover, the destruction of tropical rainforests and climate change leads to the geographical expansion of important arbovirus vectors, notably *Aedes* mosquitoes, which usually resided in tropical forest regions but are now also endemic in urban areas^1,3^. The expansion of vector distribution further fuels the upsurge of arboviral diseases. One example is the emergence of WNV in northern Europe. While WNV infection in humans usually causes mild flu-like symptoms, it can also lead to more severe (neurological) disease symptoms, including meningitis, encephalitis, and paralysis, and can ultimately lead to fatality, with immunocompromised patients and the elderly being at greater risk. There is currently no WNV vaccine available for humans^4^, and a better understanding of pathology and virulence of (novel) emerging WNV strains would aid in predicting the clinical outcome of WNV infections in humans during potential future WNV outbreaks.

WNV isolates are currently classified into 9 different genetic lineages, with isolates that are associated with severe (neurological) disease in humans grouped into 2 main lineages; lineage 1 and 2. Lineage 1 is widely dispersed around the world and has been circulating in southern Europe since the early 2000s. Lineage 2 was primarily found in sub-Saharan Africa and Madagascar, but emerged in Europe in 2004^5^ and has been circulating since. In Europe, mostly the southern regions are affected by WNV disease, however in the past few years WNV has been moving northwards, with human infections now also reported in Germany and more recently the Netherlands^6^. Due to its association with outbreaks of human neurological disease, such as a large WNV outbreak in the US in 1999^7^, lineage 1 WNV strains were previously considered to be more pathogenic compared to lineage 2. While lineage 1 is still causing sporadic outbreaks in Europe^8^, lineage 2 is currently more prevalent and likewise responsible for outbreaks of severe WNV disease in humans^9–12^, demonstrating that the circulation of lineage 2 WNV in Europe is of growing public health concern. WNV lineage 2 strains are divided into clades 2a and 2b, of which 2a is further subdivided into clusters A and B. WNV lineage 2a is currently dominant in Europe; sub-cluster A strains are found in northwest Europe, and sub-cluster B strains in southeast Europe^13^. In 2010, a WNV lineage 2a cluster B strain caused a major outbreak of neuroinvasive disease in humans in Greece, with a case-fatality rate of 17% amongst reported WNV neuroinvasive disease patients^9,14^, and cases of WNV disease in humans were also reported in several other central and south-eastern European countries including Austria, Hungary, and Italy^15^. In 2020, a new lineage 2a cluster A strain emerged in the Netherlands and is associated with the diagnosis of WNV disease in 7 individuals in the Netherlands, where there was no mortality amongst the reported patients^16–18^. How exactly this observed reduced clinical severity of the cluster A strain compares in (neuro)virulence or pathogenesis with WNV lineage 2a cluster B strains, such as the strain responsible for the outbreak in Greece and other human cases in European countries in 2010, is currently not known.

For the initial characterization and assessment of the pathogenesis and virulence of a (novel) WNV strain, in vitro and in vivo models have previously been used, including infection of cell lines^19,20^, primary cell cultures^21,22^, and wild-type mice^19,23^. Here, we assessed the pathogenicity of the 2020 isolated WNV lineage 2a cluster A strain from the Netherlands (WNV-NL20) and used a previously studied^19^ WNV lineage 2a cluster B strain from the 2010 outbreak in central and south-eastern Europe (WNV-578/10) as a reference.

## Methods

### Ethics statement

All animal procedures were performed in compliance with the Dutch legislation for the protection of animals used for scientific purposes (2014, implementing EU Directive 2010/63) and other relevant regulations. The licensed establishment where this research was conducted (Erasmus MC) has an approved OLAW Assurance # A5051-01. Animal experiments were conducted under a project license from the Dutch competent authority, and the study protocol #2010606 was approved by the institutional Animal Welfare Body.

### Cells

Vero cells (African green monkey kidney epithelial cells, ATCC CCL-81) were cultured in Dulbecco’s modified eagle’s medium (Lonza) supplemented with 10% heat-inactivated fetal calf serum (FCS; Sigma Aldrich), 100 U/ml penicillin, 100 μg/ml streptomycin, 2 mM L-glutamine (Lonza), 0.75% sodium-bicarbonate (Lonza) and 10 mM HEPES buffer (Lonza). Primary human astrocytes (Sciencell, #1800) and primary human brain pericytes (Sciencell, #1200) were cultured in culture flasks that were coated with 2 μg/cm^2^ poly-L-Lysine (Sciencell) diluted in sterile (de-ionized) water and incubated at 37 °C, 5% CO_2_ for a minimum of 1 hour to a maximum of 24 hours. Subsequently, the coated flasks were washed twice with de-ionized water prior to cell seeding. Astrocytes and pericytes were maintained in Astrocyte medium (Sciencell, #1801) and Pericyte medium (Sciencell, #1201), respectively. Primary human brain microvascular endothelial cells (BMECs; Cell systems) were cultured in flasks pre-coated with pre-warmed 1% porcine gelatin (Sigma Aldrich), incubated at 37 °C, 5% CO_2_ for a minimum of 15 minutes to a maximum of 24 hours. Gelatin was aspirated from the flasks prior to BMEC seeding in MV2 medium (Promocell, C-22121). For cell passaging, sub-confluent cell monolayers were washed with 1xPBS prior to adding 0.25% Trypsin-EDTA (Gibco) and incubated at 37 °C, 5% CO_2_ for 2–5 minutes. Trypsin was inactivated with a 1:1 mixture of FCS and culture medium, cell suspension was then spun at 120 g for 5 minutes at room temperature, resuspended in the respective medium and subsequently passaged into pre-coated flasks. For cell culture infections, astrocytes were used between passage 4–10, pericytes were used between passage 3–10, and BMECs were used between passage 7–12.

### Viruses

Passage 3 virus stocks of the WNV-NL20 (originally isolated from a *Phylloscopus collybit*, GenBank accession number OP762595.1, EVAg 010V-04311) used for both the in vitro and in vivo experiments were grown in Vero cells at 37 °C in 5% CO_2_. The WNV-578/10 (full-length infectious clone) passage 2 (grown on Vero cells) used in this study was kindly provided by Prof. Gorben Pijlman (Wageningen University & Research), originally generated by Tamás Bakonyi (Department of Microbiology and Infectious Diseases, Szent István University, Budapest, Hungary)^20^. The WNV-578/10 infectious clone exhibits similar replication kinetics as the wild-type 578/10 virus in vitro as shown previously^24^. Both the WNV-578/10 infectious clone and WNV-NL20 were sequenced as described previously^18^ to assess amino acid differences between the strains (Supplementary Table 1) and to check for any mutations that may have occurred during the passaging of the virus. The WNV-578/10 infectious clone used in this study contains the following amino acid substitutions: T100P (located in the capsid protein) and V2901A (located in the RNA-dependent RNA polymerase NS5) when compared to the original WNV-578/10 isolate (Genbank KC496015.1). The WNV-NL20 used in this study contains the following amino acid substitution: T100P (located in the capsid protein) when compared to the original WNV-NL20 isolate (Genbank OP762595.1).

### Virus titrations

Viral titers (as the median tissue culture infectious dose; TCID_50_) were determined by the end-point dilution essay on semi-confluent Vero cells seeded onto 96-well plates at a density of 2.3 × 10^4^ cells per well 24 hours prior to titration. The inoculated plates were incubated at 37 °C in 5% CO_2_, after which cytopathic effects were assessed 6 days post-infection (dpi) to determine the virus titers using the Spearman & Kärber method^25^.

### In vitro infection of primary human brain cells

For infection of the primary human brain cells, 48-well plates were coated for 1 hour with 2 μg/cm^2^ poly-L-Lysine, or for 15 minutes with 1% gelatin. Astrocytes (2.5 × 10^5^ cells/well), pericytes (2 × 10^5^ cells/well), and BMEC (1 × 10^5^ cells/well) were seeded in a 48-well plate in their respective medium 24 hours prior to infection. For infection, virus inoculum diluted to an MOI of 1 in the cell-type specific medium. The infected cells were incubated at 37 °C for 1 hour after which virus inoculum was removed and the well was washed three times with PBS before addition of cell-type specific medium. Cells were subsequently incubated at 37 °C, and 90 μl supernatant was removed and refreshed at 24, 48, and 72 hours pi (hpi) The harvested supernatants were stored at −80 °C for titration.

### Footpad WNV injection of wild-type C57BL/6 mice

Specific pathogen-free wild-type (WT) C57BL/6 age-matched female mice were obtained from Charles River Laboratories. Mice were divided into groups of two or three animals per HEPA-filtered negatively-pressured isolator filtertop-cage, and allowed to acclimatize for 7 days prior to the start of the experiment. Food and water were provided ad libitum throughout the duration of the experiment. The mice were 6–8 weeks of age the day of WNV inoculation. All invasive animal procedures were performed under inhalation anesthesia using isoflurane (4–5% for induction, 2–3% for maintenance, 800–1000 ml/min O_2_). Mice were inoculated intradermally in the footpad of the right hindleg with WNV-578/10 or WNV-NL20 at a target inoculation dose of either 10^5^ (low) or 10^6^ (high) TCID_50_ (per mouse) in a volume of 10 µl using a 0.3 mm (30 G needle) × 8 mm Micro-Fine syringe (BD 324826). Back-titration of the virus inocula revealed inoculum titers were as follows: WNV-578/10 low dose = 3.16 × 10^4^ TCID_50_/mouse, WNV-578/10 high dose = 1.47 × 10^6^ TCID_50_/mouse, WNV-NL20 low dose = 3.16 × 10^4^ TCID_50_/mouse, and WNV-NL20 high dose = 3.16 × 10^5^ TCID_50_/mouse. All mice were weighed and monitored for general health status and behavior on a daily basis. Any clinical signs of disease and aberrant behavior were documented. Every 2 days pi a 30 µl blood sample was collected from the tail vein and spun down in serum tubes (Greiner Bio-one 450533, MiniCollect Tube 0.5 ml/0.8 ml CAT Serum Sep Clot Activator) at 4000 rpm for 10 minutes to separate and collect the serum. Serum (~12 µl) was subsequently transferred to 45 µl lysis buffer (Roche, MagNA Pure 96 External Lysis Buffer 06374913001) and stored at −80 °C until further processing. On day 14 pi, or whenever a humane end-point was reached, animals were euthanized by cervical dislocation under inhalation anesthesia, and brain tissues (olfactory bulb, cerebrum, cerebellum, frontal lobe, and brainstem) were harvested and collected in 2 ml Eppendorf tubes (Greiner) containing one ceramic sphere (MP Biomedicals, 11-654-0424). Brain tissues were stored at −80 °C until further processing, then thawed once and homogenized using the Tissue Lyser II (Qiagen, 85300), spun down at 4000 rpm for 10 minutes. Subsequently, supernatants were titrated onto Vero cells as described above.

### Serum RNA isolation and real-time qPCR

Total RNA was extracted from the serum samples by transferring the serum samples to a 96-well plate and incubation with Agencourt AMPure XP magnetic beads (Beckman Coulter A63882) for at least 15 minutes. Samples were then placed onto a DynaMag-96 magnet (Invitrogen, 12027) for 3 minutes, after which the supernatant was aspirated and discarded. The beads were washed three times with 70% ethanol and air-dried for 3 minutes while kept on the magnetic block. RNA was eluted from the beads by resuspension in sterile ddH_2_O. Real-time TaqMan PCR was performed using a WNV primer/probe mix (Probe sequence TGCTGCTGCCTGCGGCTCAACCC, Forward: CCACCGGAAGTTGAGTAGACG, Reverse: TTTGGTCACCCAGTCCTCCT) diluted in TaqMan Fast Virus-1 Step Master Mix (Applied Biosystems 4444432) and ddH_2_O. The Applied Biosystems 7500 Real-Time PCR system (ThermoFisher Scientific) was used with the following program settings: 5 minutes 50 °C, 20 seconds 95 °C, 45 cycles of 3 seconds 95 °C and 30 seconds 60 °C.

### Histopathology and immunohistochemistry

During necropsies, brain tissues were collected and fixed in 4% buffered formaldehyde. Brains were dissected according to Jordan et al.^26^ to evaluate different areas. After processing and embedding in paraffin, 3–5 μm thick sections were cut and stained with Hematoxylin and Eosin (H&E) for histopathological investigation. Severity of lesions was scored according to a two-parameter scoring system considering the distribution of the lesions (0 = no lesions; 1 = focal; 2 = multifocal; 3 = diffuse) and the amount of infiltrating rounded cells and/or glial cells involved (0 = no cells; 1 ≤ 30 cells; 2 = 30–60 cells; 3 ≥ 60 cells). Scores of the two parameters were summed, and cases were allocated as follows: 2–3 = mild; 4 = moderate; 5–6 = severe. Immunohistochemistry (IHC) staining for WNV antigen was performed as previously described^19^. The presence of WNV antigen on IHC stained tissues were scored with a two-tier scoring system^27^ considering the number of positive cells in 10 high-power fields (field diameter of 0.55 mm, HPF area of 0.237 mm^2^) (0 = no positive cells; 1 = 1–10 positive cells; 2 = 11–20 cells; 3 ≥ 21 cells) and the distribution of positive cells (1 = focal; 2 = scattered; 3 = multifocal aggregates; 4 = diffuse). The scores of the two parameters were summed, and cases were allocated as follows: 0 = absent; 2–3 = low; 4–5 = moderate; 6–7 = high.

## Results

### WNV-578/10 and WNV-NL20 show similar replication kinetics in primary human brain cells

**Fig. 1:**
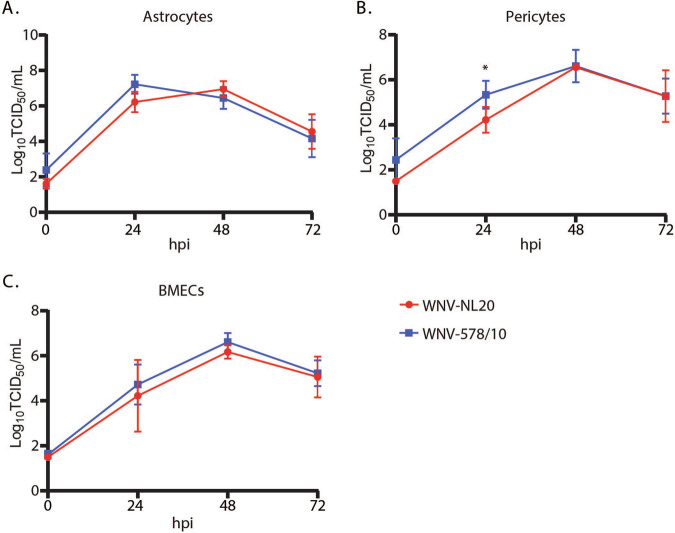
Replication kinetics of WNV-578/10 and WNV-NL20 in primary human cells of the blood-brain barrier. Primary human (**A**) astrocytes, (**B**) pericytes, and (**C**) brain microvascular endothelial cells (BMECs) were infected with either WNV-578/10 or WNV-NL20 at an MOI of 1. The supernatant was collected every 24 hours post-infection (hpi) over a total of 3 days. Significant differences at 24 hours post infection for pericytes are indicated with an asterisk (*p value = 0.0406, 2-way ANOVA, alpha 0.05). Results were pooled from two individual experiments. Error bars represent the standard deviation.

WNV-NL20 was previously shown to efficiently replicate in primary human cell cultures that constitute the blood-brain barrier^28^. Here we compared the replication kinetics of WNV-NL20 with WNV-578/10 over 3 days. Both WNV-578/10 and WNV-NL20 could infect and replicate with comparable kinetics and peak titers of 6–7 Log_10_TCID_50_/ml by 24–48 hpi in astrocytes (Fig. 1A), pericytes (Fig. 1B), and BMECs (Fig. 1C). Although WNV-578/10 produces significantly higher viral titers at 24 hpi in pericytes compared to WNV-NL20, this may be attributed to differences in residual inoculum at 0 hpi. Thus, WNV-578/10 and WNV-NL20 do not show major differences in replication kinetics in primary human brain cells in vitro.

### WNV-NL20 infection results in lethal disease in WT mice

**Fig. 2:**
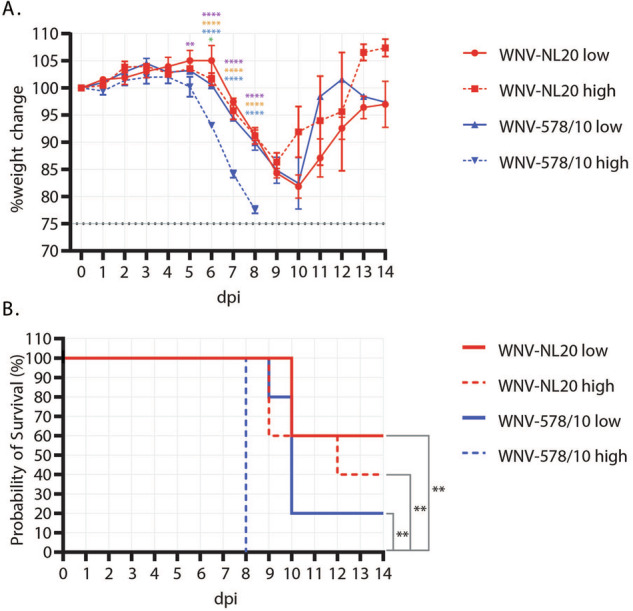
Weight loss and survival curves of C57BL/6 WT mice inoculated with different doses of WNV-578/10 (blue lines) and WNV-NL20 (red lines) via the intradermal route. Groups were inoculated at a dose of either 10^5^ (solid lines) or 10^6^ (dashed lines) TCID_50_/mouse (*n* = 5 mice per group). **A** Weight loss post-WNV-578/10 versus WNV-NL20 infection. Error bars represent the SEM. Statistical analyses over day 1–8 pi were done using a 2-way ANOVA (alpha = 0.05). Significant differences are indicated with colored asterisks as follows: WNV-NL20 low dose vs WNV-578/10 low dose (green: 6 dpi *p = 0.0168), WNV-NL20 low dose vs WNV-578/10 high dose (purple: 5dpi **p = 0.0085. 6–8 dpi ****p < 0.0001), WNV-NL20 high dose vs WNV-578/10 high dose (orange: 6–8 dpi ****p < 0.0001), and WNV-578/10 low dose vs WNV-578/10 high dose (blue: 6–8 dpi ****p < 0.0001). **B** Survival curves of infected mice. Significant differences in survival were observed between WNV-578/10 low dose and WNV-578/10 high dose, WNV-NL20 high dose and WNV-578/10 high dose, and WNV-NL20 low dose and WNV-578/10 high dose, as indicated by the asterisks (*p* value** = 0.0027) using the Log-rank (Mantel–Cox) test.

To compare the pathogenesis of the new WNV-NL20 strain with that of the previously circulating WNV-578/10 strain, C57BL/6 WT mice were inoculated in the footpad of the right hindleg with a low or high inoculation dose. Primary outcome parameters of arbovirus disease in vivo include weight loss and mortality, for which differences between the two strains and doses were observed. Mice that were inoculated with WNV-578/10 at a high inoculation dose (1.47 × 10^6^ TCID_50_/mouse) started losing weight after 4 dpi, and continued to lose weight gradually (~8% per day) until uniformly reaching the humane end-point (set at 75% of their starting bodyweight) at 8 dpi. Mice inoculated with WNV-578/10 low dose (3.16 × 10^4^ TCID_50_/mouse), WNV-NL20 high dose (3.16 × 10^5^ TCID_50_/mouse), and WNV-NL20 low dose (3.16 × 10^4^ TCID_50_/mouse) started losing weight one day later, after 5-6 dpi, and followed the same downward trend (~8% bodyweight loss per day) as WNV-578/10 high dose, until reaching humane end-points between 9-12 dpi. Surviving animals started to recover after 9 dpi (Fig. 2A). The probability of survival post-infection was lowest for WNV-578/10 high dose (0%), followed by WNV-578/10 low dose (20%), WNV-NL20 high dose (40%), and WNV-NL20 low dose (60%) (Fig. 2B). In view of the fact that the inoculated doses did not match the intended target inoculation doses, as indicated by the back-titration of inocula as stated in the material and methods section, a direct comparison between the two WNV strains can solely be done for the WNV-578/10 and WNV-NL20 ‘low dose’ groups. As such, these findings demonstrate that the WNV-578/10 strain induces similar weight loss kinetics compared to the WNV-NL20 strain. Nevertheless, while there was a trend where WNV-578/10-infected mice had a lower probability of survival (20%) compared to the WNV-NL20 strain (60%), this difference was not statistically significant.

### Neurological signs of WNV disease in WT mice

As WNV is considered a neurotropic arbovirus and known to cause neurological disease in mice^23^, all animals were monitored for neurological signs of disease post-infection, in addition to the general clinical signs of disease characterized by an abnormal gait or posture, ruffled fur, and inactivity. In the WNV-578/10 high dose group, 2 out of 5 animals showed moderate paraparesis on 8 dpi. One animal was unable to move both hindlegs, and the other animal had an impaired balance. None of the WNV-578/10 low dose group showed any neurological signs of disease. In the WNV-NL20 high dose group, 1 out of 5 animals showed moderate paraparesis on 9 dpi, presenting with dragging of its hindlegs. In the WNV-NL20 low-dose group, 1 out of 5 animals showed partial paralysis of the right hindleg on 10 dpi. These findings indicate that both WNV-578/10 and WNV-NL20 can induce neurological symptoms in WT mice post-intradermal inoculation, albeit sporadically (Supplementary Table 2).

### Higher viremia and viral load in the brains of WT mice infected with WNV-578/10 compared to WNV-NL20

**Fig. 3:**
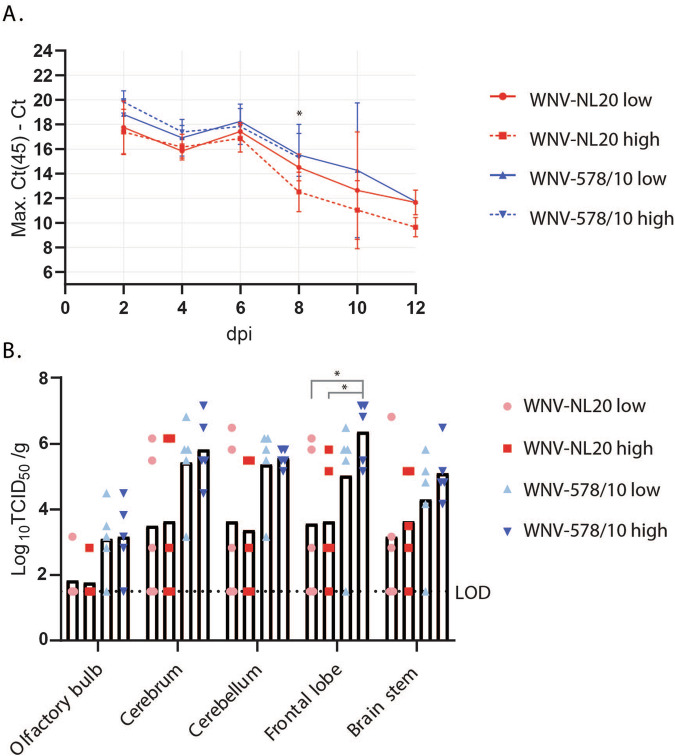
Viremia and viral load in the brain of C57BL/6 WT mice inoculated with different doses of WNV-578/10 and WNV-NL20 via the intradermal route. Statistical analyses were done using a 2-way ANOVA (alpha = 0.05) (*n* = 5 mice per group). **A** Viral load in the blood as determined by qPCR. Shown as max. Ct (45) minus the measured Ct value. Blood was sampled every 2 days post-infection up until 12 dpi, or when the humane end-point was reached. Significant differences over day 2–8 pi are indicated with asterisks as follows: WNV-NL20 high dose vs WNV-578/10 low dose 8 dpi *p = 0.0101, WNV-NL20 high dose vs WNV-578/10 high dose 8 dpi *p = 0.0186. Error bars represent the SEM. **B** Infectious virus titers in different regions of the brain of mice that were euthanized when the humane end-point was reached, as measured by virus titration. Shown as Log_10_TCID_50_ per gram of tissue. LOD = limit of detection. Significant differences between WNV-578/10 high dose vs. WNV-NL20 low dose (*p* value* = 0.0381) and WNV-578/10 high dose vs. WNV-NL20 high dose (*p* value* = 0.0449) were found for titers in the frontal lobe, as indicated by the asterisks.

As the level of viremia is generally correlated with virulence^29^, we further assessed the levels of viremia of the mice infected with different doses of WNV-578/10 and WNV-NL20 (Fig. 3A). Peak viremia was reached at 2 dpi, with both WNV-578/10 high dose and low dose reaching Ct values between ~24 and 30. A slight decrease in viremia is observed at 4 dpi, followed by a minor increase at 6 dpi for both WNV-578/10 and WNV-NL20, after which viral loads gradually decrease up to 12 dpi. No significant differences in viremia were observed between the two strains. Overall, both WNV strains showed high levels of viremia between day 2 and 6 pi.

Furthermore, as infection resulted in neurological signs, we determined the viral loads in different regions of the brain (Fig. 3B). No statistically significant differences were observed between WNV-578/10 and WNV-NL20 except for the frontal lobe, where 90% of the animals from the WNV-578/10 groups show viral titers between 5 and 7 Log_10_TCID_50_/g, but only 40% from the WNV-NL20 groups reach frontal lobe titers of ~5–6 Log_10_TCID_50_/g. However, this could again be attributed to the higher inoculation dose for the WNV-578/10 high-dose group compared to the other groups, i.e., no definitive conclusions regarding any differences in tissue titers between the two strains can be made.

### Both WNV-578/10 and WNV-NL20 are able to infect neurons and induce neuronal necrosis in brains of WT mice

**Fig. 4:**
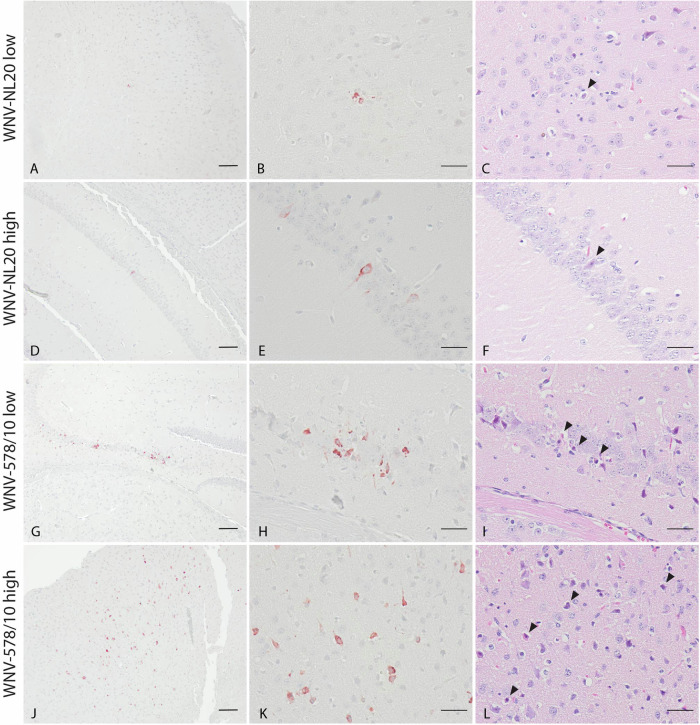
WNV antigen and neuronal necrosis in brain tissue of C57BL/6 mice infected with different doses of WNV-NL20 and WNV-578/10 via intradermal inoculation. Representative pictures of one mouse per group. Scale bars 100 μm (left column), 50 μm (middle column, right column). **A**, **B**, **J**, **K** Presence of WNV antigen in neuronal cells in the cortex. **D**, **E**, **G**, **H** Presence of WNV antigen in neuronal cells in the hippocampus. **C**, **F**, **I**, **L** Neuronal necrosis in the same area as where WNV antigen was detected (black arrowheads).

**Fig. 5:**
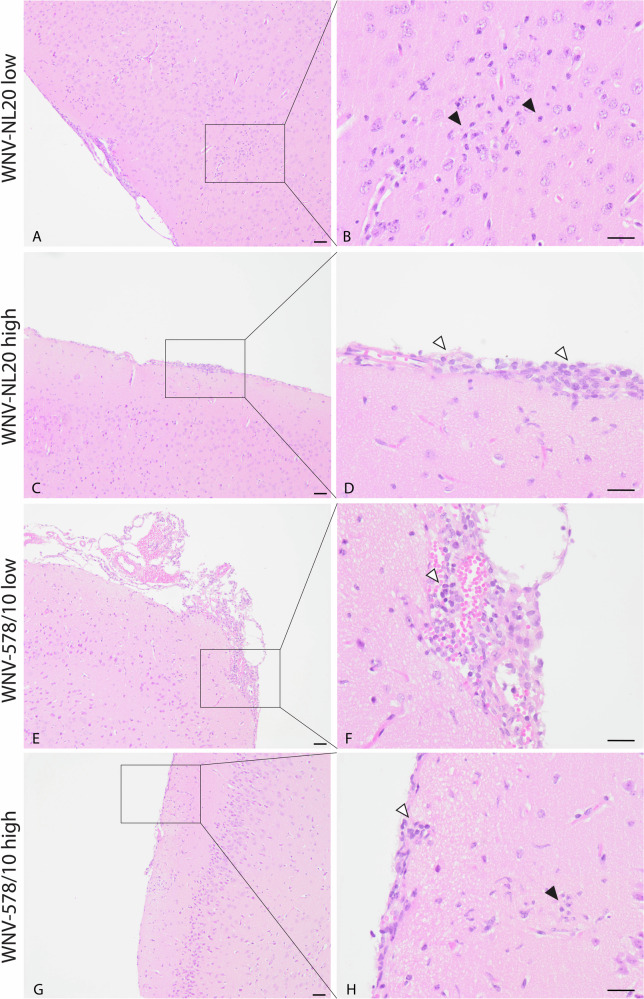
Lesions and inflammation in brain tissue of C57BL/6 mice infected with different doses of WNV-NL20 and WNV-578/10 via intradermal inoculation. Representative pictures of one mouse per group. Scalebar = 50 μm. **C**–**H** Aggregates of rounded cells in the meninges surrounding blood vessels (white arrowheads point to representative cells) and (**A**, **B**, **G**, **H**) aggregates of glial cells (black arrowheads point to representative cells) in the cortex subjacent to the affected meninges.

Since animals developed neurological signs of disease, we wanted to confirm the presence of virus antigens and histopathological lesions in the brain. Based on the immunohistochemistry staining, both WNV-NL20 and WNV-578/10 show the presence of virus antigen in the brain (Fig. 4; Supplementary Table 2). Virus antigen was observed in the cytoplasm of both grouped and scattered neurons in the cortex, hippocampus, thalamus and brainstem, as well as in the cytoplasm of Purkinje cells and neurons of the granular layer of the cerebellum (Supplementary Table 2). H&E-stained sections of the brain show neurons and Purkinje cells undergoing neuronal necrosis in the same areas that were found positive for WNV antigen (Fig. 4C, F, I, L; Supplementary Table 2). In several animals of each group, histopathological examination of H&E-stained sections of the brain revealed the presence of mild lesions represented by focal accumulation of rounded cells in the meninges surrounding the blood vessels and aggregates of glial cells (gliosis) in the cortex (Fig. 5; Supplementary Table 2). No significant differences in severity or a number of lesions were seen between animals infected with WNV-578/10 low dose, WNV-NL20 low dose, and WNV-NL20 high dose, however, the lesion score of the WNV-578/10 high dose group was higher and more uniform compared to the other groups, although this should likely be attributed to the higher inoculated dose in these animals.

## Discussion

WNV is a (re)emerging arbovirus that circulates worldwide. In Europe, WNV primarily affects the southern regions, where WNV lineage 2a strains predominantly circulate and cause sporadic outbreaks of WNV disease in humans. In recent years WNV has also been detected in northern Europe. For example, WNV was detected in the Netherlands for the first time in 2020, where a novel WNV lineage 2a strain emerged^18^. WNV lineage 2a strains that are currently circulating in Europe can be further divided into clusters A and B, with cluster A strains spreading to the West, and cluster B spreading to the south of Europe^13^. As clinical differences between genetically distinct WNV strains have been speculated upon previously^30^, we sought to assess the pathogenic properties of the newly emerged WNV lineage 2a cluster A strain and included a WNV lineage 2a cluster B strain that caused cases of WNV disease in humans in central and south-eastern Europe, including a major outbreak in Greece in 2010^9^, as a reference.

To evaluate the pathogenic potential of WNV-NL20 and WNV-578/10, we inoculated WT mice with different doses of both WNV strains. Animal models have been used previously to assess WNV pathogenesis, immune responses to infection, and neuroinvasion^31^, which are key aspects of WNV infection that cannot be measured in vitro. Because arboviruses are primarily transmitted to a new host *via* the bite of an infected mosquito, we chose to inject the virus intradermally rather than the more commonly used intraperitoneal or subcutaneous injection route. In this regard, the virus has to overcome the first immunological barrier during initial replication in the skin, after which the virus disseminates to the draining lymph nodes and distinct organs^32^. Bypassing this important aspect of arbovirus transmission, for example, by injecting intraperitoneally, may result in losing the detection of potential variation in pathogenesis between WNV strains at the initial stages of infection. Importantly, efficient early peripheral replication is most likely linked to the efficiency by which neurotropic arboviruses invade the brain and induce mortality of the host^33,34^. Intradermal inoculation of both WNV-578/10 and WNV-NL20 in WT mice resulted in weight loss, development of neurological signs, detectable viremia and infectious virus titers in the brain, and mortality in all groups. Development of neurological signs seems to be associated with aggregates of glial cells in the cortex, similar to previously published data^35–37^, although causation was not proven in the current study. The 0% survival of the WNV-578/10 high dose group corroborates earlier findings^20^, however both the inoculation route and dose differ from our study. Overall, no significant differences between WNV-NL20 and WNV-578/10 in both in vitro and in vivo infections were found. The high viral titers in the brain of WNV-infected mice could be associated with the observed viremia. WNV is thought to use various hematogenous routes of neuroinvasion across the blood-brain barrier^38^, therefore the development of (high) viremia likely aids in neuroinvasion. The observed biphasic viremia suggests initial WNV replication at the inoculation site, resulting in a primary viremia, followed by dissemination to distinct organs for replication and correspondingly inducing secondary viremia. While not previously reported, these kinetics are in line with the observation that WNV is initially only detected at the inoculation site (the skin), followed by detection in peripheral organs and subsequent detection in the brain^35^. However, a serial-sacrifice study would be required to study whether the efficiency of early replication in the dermis indeed facilitates WNV neuroinvasion and gain a better understanding of potentially different time scales at which WNV strains end up in the brain. Similar to the in vivo data, no differences in replication kinetics were observed in vitro, where both WNV strains showed similar replication kinetics and peak titers in primary human astrocytes, pericytes, and brain microvascular endothelial cells.

In conclusion, our findings confirm that WNV-NL20 is neurovirulent in mice and, while keeping in mind the limitations of using animal models to study virulence and viral pathogenesis in humans^39^, suggest that this WNV lineage 2a cluster A strain does not differ in pathogenicity compared to WNV lineage 2a cluster B strains. However, the lack of significant difference between WNV-NL20 and WNV-578/10 in vivo does not reflect the WNV (cluster B) outbreak of 2010 in Greece (17% mortality among reported patients)^9^ when compared to the WNV (cluster A) outbreak of 2020 in the Netherlands (no mortality among reported patients)^16^. Given the low number of cases in the Netherlands, further surveillance of humans is required in order to attribute the severity of the disease to the different WNV clusters. Since WNV-578/10 has been associated with outbreaks of severe WNV disease in humans, we should remain vigilant for the detection of human neurological disease of unknown causes.

## Supplementary information

Supplementary information

## Data Availability

The datasets used and/or analysed during the current study are available from the corresponding author upon reasonable request.

## Code availability

N/A.
