# Supplementary material for: In vitro and in vivo characterization of a novel West Nile virus lineage 2 strain
Source: Npj Viruses. 2024 Nov 25;2:61. doi: 10.1038/s44298-024-00070-0 (PMC11721649; doi:10.1038/s44298-024-00070-0)
Supplement: Supplementary file 1 — Supplementary information [file 44298_2024_70_MOESM1_ESM.pdf]

Supplementary

Supplementary Table 1. Protein-amino acid substitutions in WNV-NL20 (passage 3 on Vero) compared to the WNV-578/10 infectious clone (passage 2 on Vero) used in this study. Single-letter abbreviations for amino acid residues are as follows: A, Alanine; C, Cysteine; D, Aspartic acid; E, Glutamic acid; F, Phenylalanine; G, Glycine; H, Histidine; I, Isoleucine; K, Lysine; L, Leucine; M, Methionine; N, Asparagine; P, Proline; Q, Glutamine; R, Arginine; S, Serine; T, Threonine; V, Valine; W, Tryptophan; and Y, Tyrosine.

WNV-NL20	WNV-578/10	Location on WNV polyprotein
L284M	L	Membrane protein **M**
R383K	R	Envelope protein **E**
T447A	T	Envelope protein **E**
I449T	I	Envelope protein **E**
A990S	A	Non-structural protein **NS1**
Y1262C	Y	Non-structural protein **NS2A**
V1355M	V	Non-structural protein **NS2B**
V1405I	V	Non-structural protein **NS2B**
I1493V	I	Non-structural protein **NS3**
P1754H	P	DEAD-domain of serine protease **NS3**
S2284N	S	Non-structural protein **NS4B**
G2287S	G	Non-structural protein **NS4B**
R2293K	R	Non-structural protein **NS4B**
A2322T	A	Non-structural protein **NS4B**
A2827T	A	RNA-dependent RNA polymerase **NS5**
A2901V	A	RNA-dependent RNA polymerase **NS5**
G3159R	G	RNA-dependent RNA polymerase **NS5**

Supplementary Table 2. Combined overview of brain titers, day of sacrifice/euthanasia, neurological signs, immunohistochemistry scoring, lesion scores and brain lesions of all C57BL/6 WT mice inoculated intradermally with WNV-578/10 or WNV-NL20 at a high or low dose. EE = End of Experiment (14 days post-infection). High brain titers indicate 10^5-10^7 $TCID_{50}$/g tissue, low brain titers indicate 10^2-10^3 $TCID_{50}$/g tissue. DOS = Day of sacrifice. Immunohistochemistry scoring system; # of positive cells: 0 = no signal, 1 = 1-10 labelled cell(s), 2 = 2-20 labelled cells, 3 = >21 labelled cells. Signal distribution in tissue: 1 = focal, 2 = scattered, 3 = multifocal/patchy, 4 = diffuse. Total grade: 0 points = no signal, 2-3 points = mild, 4-5 pints = moderate, 6-7 points = severe. Lesion/inflammation scoring system (H&E staining); Distribution of lesions: 0 = absent, 1 = focal, 2 = multifocal, 3 = diffuse. Number of infiltrating rounded/glial cells: 0 = absent, 1 = <30 cells, 2 = 30 to 60 cells, 3 = >60 cells. Total score: 2-3 = mild, 4 = moderate, 5-6 = severe.

					IHC scoring					H&E scoring			
**Group**	**Animal#**	**Brain titers**	**DOS**	**Neurological signs**	**+ve cells**	**Distribution**	**Grade**	**Cells**	**Location**	**Infiltrating cells**	**Distribution**	**Grade**	**Brain lesions**
WNV-NL20 low dose	1	High	10	-	2	3	Moderate	Neurons; Purkinje cells	Hippocampus, cortex, basal nuclei, cerebellum	0	0	-	Focal area of gliosis in the hippocampus; hyperaemia in the cortex; neuronal necrosis Purkinje cells
	2	-	EE	-	0	0	No signal	-	-	1	2	Mild	Focal area of increased cellularity in the meninges
	3	-	EE	-	0	0	No signal	-	-	2	1	Mild	Few mild aggregates of glial cells in the cortex; focal area of increased cellularity in the meninges
	4	High	10	Right hind-leg partial paralysis	3	3	Severe	Neurons; Purkinje cells	Hippocampus, cortex, basal nuclei, cerebellum, brainstem	0	0	-	Neuronal necrosis; multifocal gliosis in the cortex and thalamus
	5	Low	EE	-	1	1	Mild	Neurons	Cortex	0	0	-	Mild gliosis in the cortex
WNV-NL20 high dose	6	Low	EE	-	1	1	Mild	Purkinje cells	Cerebellum	1	1	Mild	Focal area of increased cellularity in the meninges
	7	High	9	-	1	2	Mild	Neurons	Cortex	0	0	-	Focal neuronal necrosis
	8	Low	12	-	0	0	No signal	-	-	0	0	-	-
	9	High	9	Slight dragging of hind-feet	1	2	Mild	Neurons; Purkinje cells	Cortex, hippocampus, brainstem, cerebellum	1	1	Mild	Focal area of increased cellularity in the meninges of the brainstem; neuronal necrosis of Purkinje cells Focal; neuronal necrosis hippocampus focal
	10	Low	EE	-	1	2	Mild	Neurons	Cortex	0	0	-	Focal gliosis cortex and basal nuclei

**WNV-578/10 low dose**	11	High	10	-	3	2	Moderate	Neurons; Purkinje cells	Cortex, hippocampus, cerebellum, brainstem	0	0	-	Increased cellularity in the meninges; neuronal necrosis (focal) hippocampus and cerebellum; focal gliosis in the cortex
	12	High	10	-	1	2	Mild	Neurons; Purkinje cells	Cortex, hippocampus, cerebellum, brainstem	0	0	-	Focal gliosis cortex
	13	Low	EE	-	0	0	No signal	-	-	2	1	Mild	Multifocal areas of increased cellularity in the meninges
	14	High	10	Tremors	1	2	Mild	Neurons; Purkinje cells	Cerebellum, brainstem	0	0	-	Focal gliosis; neuronal necrosis
	15	High	9	-	1	2	Mild	Neurons	Hippocampus, cortex	0	0	-	Neuronal necrosis
**WNV-578/10 high dose**	16	High	8	Paralysed hind legs, extremely tilted to the left	3	3	Severe	Neurons; Purkinje cells; glial cells	Brainstem, cerebellum, cortex, hippocampus, thalamus, basal nuclei	2	2	Moderate	Gliosis; neuronal necrosis; haemorrhage (focal) brainstem; focal area of increased cellularity in the meninges
	17	High	8	-	2	3	Moderate	Neurons	Hippocampus, cortex, olfactory bulb	2	2	Moderate	Focal necrosis (malacia); focal area of increased cellularity in the meninges
	18	High	8	-	2	3	Moderate	Neurons	Hippocampus, cortex, thalamus, basal nuclei, brainstem	2	2	Moderate	Focal gliosis; neuronal necrosis
	19	High	8	Trouble finding balance	2	2	Moderate	Neurons; Purkinje cells	Basal nuclei, hippocampus, cerebellum	1	1	Mild	Perivascular cuffs; neuronal necrosis; focal area of increased cellularity in the meninges
	20	High	8	-	1	1	Mild	Neurons	Brainstem	1	1	Mild	Focal area of increased cellularity in the meninges
